# MG1141A as a Highly Potent Monoclonal Neutralizing Antibody Against SARS-CoV-2 Variants

**DOI:** 10.3389/fimmu.2021.778829

**Published:** 2021-11-18

**Authors:** Sua Lee, Shina Jang, Jihoon Kang, Soo Bin Park, Young Woo Han, Hyemi Nam, Munkyung Kim, Jeewon Lee, Ki Joon Cho, Jeonghun Kim, Miyoung Oh, Jihye Ryu, Jong Hyeon Seok, Yunhwa Kim, Jee-Boong Lee, Man-Seong Park, Yong-Sung Kim, Hosun Park, Dong-Sik Kim

**Affiliations:** ^1^ Department of Protein Engineering, Mogam Institute for Biomedical Research, Yongin, South Korea; ^2^ Department of Infectious Disease Research, Mogam Institute for Biomedical Research, Yongin, South Korea; ^3^ Department of Target ID & Assay Development, Mogam Institute for Biomedical Research, Yongin, South Korea; ^4^ Department of Microbiology, Institute for Viral Diseases, Korea University College of Medicine, Seoul, South Korea; ^5^ Department of Translational Research, Mogam Institute for Biomedical Research, Yongin, South Korea; ^6^ Department of Microbiology, College of Medicine, Yeungnam University, Daegu, South Korea; ^7^ Department of Molecular Science and Technology, Ajou University, Suwon, South Korea

**Keywords:** MG1141A, SARS-CoV-2, monoclonal antibody, outbreak, spike protein

## Abstract

Since the coronavirus disease outbreak in 2019, several antibody therapeutics have been developed to treat severe acute respiratory syndrome coronavirus 2 (SARS-CoV-2) infections. Antibody therapeutics are effective in neutralizing the virus and reducing hospitalization in patients with mild and moderate infections. These therapeutics target the spike protein of SARS-CoV-2; however, emerging mutations in this protein reduce their efficiency. In this study, we developed a universal SARS-CoV-2 neutralizing antibody. We generated a humanized monoclonal antibody, MG1141A, against the receptor-binding domain of the spike protein through traditional mouse immunization. We confirmed that MG1141A could effectively neutralize live viruses, with an EC_50_ of 92 pM, and that it exhibited effective Fc-mediated functions. Additionally, it retained its neutralizing activity against the alpha (UK), beta (South Africa), and gamma (Brazil) variants of SARS-CoV-2. Taken together, our study contributes to the development of a novel antibody therapeutic approach, which can effectively combat emerging SARS-CoV-2 mutations.

## Introduction

The COVID-19 pandemic has caused more than 100 million infections and over 2 million deaths worldwide since its outbreak in 2019 (1). Not only has the pandemic caused incalculable damage to human health, but it has also impacted the world economically and socially. SARS-CoV-2, the causative agent of COVID-19, is a member of the beta-coronavirus family. Members of this family of viruses were also responsible for the SARS-CoV outbreak in 2004 and the MERS-CoV outbreak in 2015, which caused severe symptoms in patients. The sequence homology between SARS-CoV-2 and SARS-CoV and their spike (S) proteins is estimated to be 76% (2). The S protein is a glycoprotein that forms a homotrimeric structure, and its receptor-binding domain (RBD) binds to human angiotensin-converting enzyme 2 (ACE2), which regulates both cross-species and human-to-human transmissions of SARS-CoV (3). Therefore, S protein is the primary target for therapeutic agents and vaccines as it directly modulates host cell infection (4). Currently, antibody treatments, small molecules, and intravenous immunoglobulins are being developed as therapeutic agents to treat SARS-CoV infections; several antibody treatments are currently in clinical trials, and three of them have received emergency-use authorization from the FDA (5–7).

Antibody therapeutics exhibit several antiviral functions that are dependent on the antibody structure. Antibodies consist of two main structural regions: the Fab region binds to the target antigen, and the Fc region interacts with Fc-receptors on innate immune cells to mediate downstream effector functions. Therefore, antibodies block viral entry into host cells and enhance the clearance of viruses and infected cells through Fc-mediated effector functions such as antibody-dependent cellular cytotoxicity (ADCC) or antibody-dependent cellular phagocytosis (ADCP). The antibody neutralizes SARS-CoV-2 by binding to the RBD of the viral S protein and blocking its binding to the host cell receptor ACE2 (8–10). However, SARS-CoV-2 variants with S protein mutations have recently emerged that exhibit enhanced transmission efficiency. The initial S protein mutation was a D614G mutation in the G clade, which was reported in February 2020. The D614G mutation promoted the “standing-up” conformation of the RBD, wherein the key epitopes of the domain are more accessible, compared with those of wild type (WT) SARS-CoV-2 (11, 12). The affinity of the trimeric S protein toward ACE2 was slightly increased due to this mutation (13). In addition, the D614G mutation improved the stability of the S protein, as well as replication and fitness of the virus (14). All the SARS-CoV-2 variants detected in 2021 possess the D614G mutation (15). Although the D614G mutation increases infectivity by altering the conformational state of the viral proteins, it does not reduce the efficacy of antibody therapy, immune antibodies, and vaccines in humans who have undergone treatment after an initial WT infection (11, 14, 16). However, recent mutations, such as N501Y in the alpha variant (United Kingdom, lineage B.1.1.7), K417N, E484K, and N501Y in the beta variant (South Africa. B.1.351), and K417T, E484K, and N501Y in the gamma variant (Brazil, B.1.1.28), are located in the receptor-binding motif (RBM), which directly binds to ACE2 in RBD (17, 18). N501Y increases the affinity of RBD to ACE2 (19, 20), and E484K, in particular, converts the charge on the flexible loop region of the RBD to form a new favorable contact point. As a result of the E484K mutation, N/T417 is in proximity to ACE2 and is expected to induce mutations in amino acids that can increase the binding strength to the receptor (21). These mutations increase the infectivity of the virus by increasing the affinity of SARS-CoV-2 toward ACE2. In addition, as the RBM is a major epitope to which a therapeutic neutralizing antibody binds, mutations in the RBM reduce the affinity of neutralizing antibodies, thereby reducing the neutralization efficiency (22). These mutations have also been reported to reduce the efficacy of vaccines currently under development (20, 23, 24). Accordingly, there is a need to develop therapeutic antibodies that are effective against SARS-CoV-2 variants in the future.

The use of antibody therapy is limited as it can only be used to treat patients with mild and moderate SARS-CoV-2 infections (5, 7, 25, 26). Although antibody therapy is effective in neutralizing the virus, it does not alleviate the inflammation that occurs in patients with severe infections (25). The inflammation caused by SARS-CoV-2 has not been clearly defined; an inflammatory signal is triggered by virus cleavage after the ACE2-expressing airway epithelial cells are infected by SARS-CoV-2. SARS-CoV-2 enters the target cell by binding to ACE2 *via* the RBD of its S protein, and human proteases act as entry activators. The viral membrane protein is expressed on target cells and activates inflammation-associated signaling pathways (27–29). Therefore, antibodies that exhibit a high Fc-mediated effector function may effectively remove the virus and infected cells, thereby attenuating the progress of the disease.

In this study, we developed an antibody that effectively neutralizes the recently detected SARS-CoV-2 variants and has an efficacy equivalent to that of previously developed antibodies. Our results present new possibilities for antibody therapeutics using these novel antibodies.

## Materials and Methods

### Recombinant Protein Expression and Purification

Genes encoding the ecto-domain (residues 13–1,202 aa) and the RBD (residues 319–541 aa) of the SARS-CoV-2 spike glycoprotein were synthesized and cloned into the pCIW mammalian expression vector with a C-terminal 6x histidine tag. Genes encoding RBD mono-Fc and human ACE2 mono-Fc [obtained from Prof. Jason S. McLellan, University of Texas (30)] were cloned into the pCIW vector. The pCIW mammalian vectors were transfected into Expi293F™ cells (Cat. no. A1435101). All the steps for expression analysis were performed according to the manufacturer’s instructions. After 5–6 days of cell cultivation, the cells were harvested by centrifugation, and the supernatant was passed through a 0.22 μm filter to remove cell debris. For His-tag purification of recombinant proteins, the Ni-NTA resin was added to the supernatant. MabSelect Xtra (Cat. no. 17-5269-02, GE Healthcare) was used as a resin for protein-A purification of Fc-fused proteins. All purification procedures were performed according to the manufacturer’s instructions. The buffer of eluted proteins was changed with phosphate-buffered saline (PBS) using Zeba Spin Desalting Columns (Cat. no. 0089892). Protein concentration was quantified using a Nanodrop 2000C spectrophotometer (Thermo Scientific) and using SDS-PAGE under non-reducing and reducing conditions.

### Mouse Immunization

Mouse immunization experiments were approved by the Institutional Animal Care and Use Committee of GC Pharma (#GC-20-006A). Female BALB/c mice (Orientbio, Korea), aged 6 weeks, were immunized with an emulsion mixture of recombinant 100 µg of RBD mono-Fc and complete adjuvant (Freund’s Complete Adjuvant, Cat. no. F5881, Sigma-Aldrich). The mice were primed by intraperitoneal injection and administered booster doses on days 14 and 21. On days 28, 29, and 30, the mice received final booster doses of 50 µg of RBD mono-Fc without adjuvant *via* intravenous injection. On day 31, splenocytes were harvested from the sacrificed mice to construct a phage display library. Immune sera were collected from the mice prior to each immunization, and antibody titers in the serum were evaluated by ELISA. Briefly, 100 ng of RBD mono-Fc was coated onto 96-well ELISA plates and incubated at 4°C overnight. The next day, the plates were washed four times with 200 µL of PBST (0.05% Tween 20 in PBS) and blocked with 200 µL of 3% bovine serum albumin in PBS (BSA/PBS) for 1 h at room temperature (RT). The plates were then washed four times with PBST. Pre-immune and immune anti-sera isolated from mice at a 1:10,000 dilution in 3% BSA/PBS were added to the RBD mono-Fc coated plates and incubated for 1 h at RT. The plates were washed four times and antibody titers were detected using anti-mouse antibodies conjugated with horseradish peroxidase (HRP; Cat no. A0168, Sigma-Aldrich) and incubated for 1 h at RT. The plates were washed four times with PBST and developed with tetramethylbenzidine (TMB) solution/H_2_SO_4_ stop solution (Cat. no. 50-76-03, KPL). Finally, the absorbance was measured at a wavelength of 450 nm on an ELISA plate reader (Perkin Elmer).

### Phage Display Library Construction

Splenocytes were harvested from RBD-immunized mice. RNA extraction from fresh splenocytes was performed using the QIAGEN RNeasy Plus Mini Kit (Cat. no. 74104, Qiagen). cDNA was obtained by reverse transcription using the mRNA as a template (SuperScript IV first-strand synthesis system, Cat. no. 18090010, Invitrogen). The cDNA was then amplified and used in the 5ʹ RACE system (Cat. no. 634858, Clonetech) (31). The cDNA library was constructed according to the manufacturer’s instructions. The concentration of cDNA was quantified using a Qubit4 fluorometer (Thermo Scientific). To construct the phage scFv library, a heavy-chain variable domain and two variable domains of the light chain Vκ and Vλ were amplified from cDNA using a mouse germline primer set (32). The amplified genes of heavy- and light-chain variable domains were adjusted to an equal molar ratio and used as templates with a linker [(G4S)3] in an overlapping PCR reaction. The assembled VH-Vκ/Vλ vector was cloned into the pComb3XTT phagemid vector. Five vials of 1 µg phagemid were transformed into *Escherichia coli* XL1-blue cells (electroporation-competent cells; Cat. no. 200228, Stratagene) separately at 2500 V with an electroporator (Bio-Rad). The size of the library was calculated from colony counting on plates by serial dilution.

### Phage Display Library Screening

RBD-His (1 µg in PBS) was coated onto a polystyrene immuno-tube (No. 444202, Nunc) and incubated at 4°C overnight. The RBD-coated immuno-tubes were blocked with 3% BSA/PBS at 37°C for 2 h. The phage library (10^13^ CFU/mL in 1% BSA/PBS) was then added to the tube and incubated at 37°C for 2 h. The tube was washed 4–6 times with PBST, and then the bound phages in the immuno-tube were eluted with 1 mL of glycine-HCl (pH 1.5) at RT for 10 min, and 150 µL of 2 M Tris-Cl (pH 8.8) was immediately added for neutralization. The neutralized phages were mixed with 10 mL of XLI-Blue (OD_600_ = 0.4) at RT for 30 min. The infected cells were added to 90 mL of super broth (SB 35 g/L tryptone, 20 g/L yeast extract) containing 200 µL of tetracycline (50 mg/mL) (Cat. no. E709-1, Amresco) and 100 µL of carbenicillin (100 mg/mL carbenicillin; Cat. no. C1389, Sigma-Aldrich) and incubated with rotations at 220 rpm at 37°C for 1 h. The cells were infected with M13KO7 helper phage (> 10^11^ pfu/mL; Cat. no. N0315S, NEB) and incubated at 220 rpm at 37°C for 1 h. The cells were treated with 100% kanamycin (50 mg/mL; Cat. no. E713-1, Amresco) and cultured overnight at 37°C and 220 rpm. The next day, for the rescue phage library, the supernatant was harvested by centrifugation at 6,000 rpm for 15 min. Next, 5X polyethylene glycol/NaCl was added to the supernatant at a 1× dilution and incubated on ice for 1 h. The supernatant was discarded after centrifugation. The precipitate was resuspended in 1% BSA/PBS, and the supernatant was harvested by centrifugation and used in the subsequent panning process. The procedure described above was repeated three times. Single colonies were randomly collected from the third round of panning the library and inoculated into a deep 96-well culture plate, followed by overnight cultivation. Harvested cells in the plate were stored at −70°C for further use. The supernatant of the single clones was bound to RBD-coated ELISA plates. Finally, the plates were read on an ELISA plate reader (Perkin Elmer), and clones with high OD values were selected as positive clones for sequencing.

### Antibody Expression and Purification

Anti-SARS-CoV-2 scFvs were converted into the human IgG1 format by subcloning into pCIW. Expi293F cells were prepared in 30 mL of Expi293 expression medium (Cat. no. A1435101, Gibco) at a concentration of 2.5 × 10^6^ cells/mL (37°C, 8% CO_2_, 125 rpm, viability ≥ 95%). These cells were transfected with a DNA mixture composed of 30 μg of DNA (pCLW-anti-SARS-CoV-2 heavy chain: 15 μg, pcIw-anti-SARS-CoV-2 light chain: 15 μg), OptiPro SEM medium (Cat. no. 12309019, Gibco), and ExpiFectamine 293 reagent (Cat. no. A14524, Gibco). Cultivation and protein-A purification were performed as described previously.

### Competition ELISA Assay

For ELISA, 30 nM of ACE2 mono-Fc was coated per well on a 96-well ELISA plate and incubated at 4°C overnight. The next day, the wells were blocked with 3% BSA/PBS for 1 h, and the mixture of spike protein and antibodies was added and incubated for 1 h at RT. The mixture was mixed with the spike protein (10 nM) and antibodies (at a concentration ranging from 0.5 pM to 500 nM). Plates were washed with 0.05% PBST and rabbit anti-SARS-CoV-2 spike antibody (Cat. no. 40589-T62, Sino Biological) was added at a 1/1,000 dilution. After 1 h of incubation at RT, the plates were washed with 0.05% PBST and goat anti-rabbit IgG-HRP (Cat. no. 31460, Invitrogen) was used as a secondary antibody. After 1 h of incubation, the plates were washed with 0.05% PBST. After 5 min of incubation with 50 µL TMB solution, 50 µL of stop solution was added. Absorbance was measured at 405 nm. The experiments were performed in triplicates.

### Antibody Binding Kinetics of Antibodies Toward S Protein

The binding kinetics of anti-S protein antibodies were determined using a Biacore T-200 biosensor (Cytiva). The antibodies were diluted with HBS-EP buffer (Cat. no. BR100826, Cytiva) (running buffer) to make up a concentration of 10 µg/mL and captured at a flow rate of 10 µL/min until Rmax reached 200 Ru on an SA chip (Cat. no. BR100530, Cytiva). S protein serially diluted in HBS-EP buffer was run on the antibody-captured SA chip at a concentration of 0.3125–20 nM at a flow rate of 30 µL/min for 3 min for association and 30 min for dissociation. Next, 10 mM glycine-HCl (pH 1.5) (Cat. no. BR100354, Cytiva) was run for 30 s to wash the S protein that was bound to the antibody. A sensorgram of antibodies was analyzed using Biacore evaluation software, and the value in the analysis concentration range that had the lowest chi-square value was selected. Four or more concentrations were selected at this time.

### Antibody Binding Analysis Against RBD Variants

Antibody binding affinity for alpha (UK, Cat. no. 40592-V08H82, Sino Biological), beta (South Africa, Cat. no. 40592-V08H85, Sino Biological), and gamma (Brazil, Cat. no. 40592-V08H86, Sino Biological) (**Supplementary Table 1**) variants were measured using octet QKe. RBD variants (5 µg/mL) were immobilized on the Ni-NTA biosensor for 150 s and reacted with 0.031–125 nM antibody for 150 s. The reacted biosensors were dipped into the dissociation well and dissociation was recorded for 300 s. The association and dissociation steps were analyzed using data analysis software. Background correction was performed using a reference biosensor in which the antibody was not bound. The analysis model was set to a 1:1 binding model, and fitting data were secured by global fitting.

### Pseudovirus Production

Pseudoviruses bearing the full-length spike protein of SARS-CoV-2 and variants carrying a firefly luciferase reporter gene were produced in Lenti-X 293T cells (Cat. no. 632160, Takara). Briefly, 10 µg of psPAX2 (plasmid no. 12260, Addgene), 10 µg of pLVX-Luciferase and 10 µg of SARS-CoV-2 S (codon-optimized) expression plasmids were co-transfected with polyethylenimine (mass ratio of 1:2) (Cat. no. 23966, Polysciences) in Lenti-X 293T cells. At 72 h post-transfection, the supernatant was centrifuged for 15 min at 2200 rpm followed by filtration *via* 0.45 µm filters and stored at −70°C for further use. Pseudovirus titers were determined by measuring the relative luciferase units.

### Neutralization Assay With SARS-CoV-2 Pseudovirus

The ACE2-HEK293 stable cell line (Cat. no. M00770, Genscript) was maintained in Dulbecco’s modified Eagle medium (DMEM; Cat. no. 11995-065, Gibco) supplemented with 10% heat-inactivated fetal bovine serum (FBS; Cat. no. 16000-044, Gibco), an antibiotic–antimycotic cocktail (Cat. no. 15240, Gibco), and 2 mg/mL hygromycin B (Cat. no. 10687010, Thermo Fisher Scientific). A 96-well flat bottom plate (Cat. no. 167008, Thermo Scientific Nunc) was coated with 10 μg/mL collagen I (Cat. no. A1048301, Thermo Fisher Scientific) and incubated at 37°C in an atmosphere containing 5% CO_2_ for 4 h. ACE2-HEK293 cells were inoculated into a collagen-coated 96-well flat bottom plate at 1 × 10^5^ cells/well in DMEM (Cat. no. 11995-065, Gibco), in which 10% heat-inactivated FBS and antibiotic–antimycotic cocktail was added 24 h prior to the assay. Antibodies were serially diluted 3-fold with DMEM containing 2% heat-inactivated FBS and antibiotic–antimycotic cocktail (this will be referred to as ‘infectious media’), for an 11-point dilution curve in the assay beginning at 10 μg/mL (66.67 nM). SARS-CoV-2 pseudoviruses were diluted 1:10 in the infectious media. Antibody dilutions were mixed 1:1 with 1/10 diluted pseudovirus and incubated at 37°C in an atmosphere containing 5% CO_2_ for 1 h. The supernatant was removed from ACE2-HEK293 cells and replaced with 50 μL of antibody–pseudovirus mixture. The cells were then incubated at 37°C in an atmosphere containing 5% CO_2_ for 1 h. Fifty microliters of infectious media was added to the cells, and the cells were further incubated at 37°C in an atmosphere containing 5% CO_2_ for 48 h. The cells were lysed with 5X reporter lysis buffer (Cat. no. L3971, Promega) and relative luciferase activity was determined using a luciferase assay system (Cat. no. E1501, Promega) and a luminometer (Cat. no. GM2010, Promega). Relative luciferase units were converted to percent neutralization and plotted with a non-linear regression curve fit in Prism Software (GraphPad, Prism 8.0).

### Plaque Assay

A plaque assay was performed using Vero cells to quantify infectious virions. Briefly, we infected Vero cells with 10-fold serial dilutions of GH clade virus (BetaCoV/South Korea/KUMC45/2020) isolated from a patient’s plasma at 37°C in 5% CO_2_ for 1 h. Subsequently, the cells were washed three times with PBS after infection, and then 2 mL of an overlay medium was added. The overlay medium was composed of 2% agar and 1 μg/mL l-(tosylamido-2-phenyl) ethyl chloromethyl ketone (TPCK)-treated trypsin (Cat. no. 857254, Sigma-Aldrich). The infected cells were stained with crystal violet to count the number of plaques after 72 h.

### Authentic SARS-CoV-2 Plaque Reduction Neutralization Test (PRNT)

Monoclonal antibodies that were serially diluted two-fold were mixed with an equal amount of virus suspension containing 100 plaque-forming units at 37°C for 1 h. The virus–antibody mixtures were inoculated into Vero cells to measure the PRNT_50_. The PRNT_50_ titer was calculated as the highest serum dilution that showed a 50% reduction in the number of viral plaques in comparison with that of a PBS-treated control. To compare with PRNT_50_ titer, neutralizing dilution (ND_50_) titer was determined as well, which was calculated using the Spearman–Karber formula.

### Generation of a Cell Line That Expresses the Spike Protein of SARS-CoV-2

To generate a stable cell line that expresses the spike protein of SARS-CoV-2 (HT1080-S), the pcDNA3.1-spike protein of SARS-CoV-2 was transfected into HT1080 cells using Lipofectamine 3000 Transfection Reagent (Cat. no. L3000075, Invitrogen), and the transformants expressing SARS-CoV-2 S protein were selected with 1.5 mg/mL geneticin (Cat. no. 10131-035, Gibco). The expression of SARS-CoV-2 S protein was confirmed using flow cytometry (FACS LSR Fortessa, BD Biosciences) using REGN10933, an anti-SARS-CoV-2 antibody.

### Measurement of Fc-Mediated Effector Functions

For the ADCC assay, HT1080-S cells served as target cells and Jurkat-NFAT-Luc/FcγRIIIa served as effector cells. They were incubated with titrated concentrations of antibodies for 15 min (Cat. no. G7018, Promega) (E:T ratio = 10:1). After incubation at 37°C in an atmosphere containing 5% CO_2_ for 6 h, Bio-Glo luciferase assay reagent was added, and luminescence was measured according to the manufacturer’s instructions. For the ADCP assay, HT1080-S cells were incubated with titrated concentrations of antibodies for 15 min and Jurkat-NFAT-Luc/FcγRIIa-H cells (Cat. no. G9995, Promega) were added as effector cells (E:T ratio = 5:1). Incubation and luminescence measurements were performed as described previously.

### Epitope Binning

Epitope binning was performed in three steps using the BLI system (Octet Qke): step 1 involved antigen immobilization, step 2 involved 1^st^ antibody binding, and step 3 involved 2^nd^ antibody binding. Between each step, a baseline step with a duration of 60 s was set to check the baseline signal. In step 1, 20 µg/mL purified recombinant SARS-CoV-2 Spike protein RBD-His was immobilized in a pre-hydrated anti-penta-His biosensor at 1,000 rpm for 200 s. In step 2, 37.5 µg/mL of different 1^st^ binding Abs were bound for 300 s. In step 3, one type of the 2^nd^ antibody (18.75 µg/mL) was bound for 150 s to check the degree of self-binding and competition. The degree of competition was defined as a percentage. The case in which only the 2^nd^ antibody was bound to the immobilized antigen was defined as 100%. Relative to this reference, a binding level of < 33% was defined as complete competition, the level between 33% and 66% was defined as intermediate competition, and a level of > 66% was defined as non-competition. Similar experiments were conducted to confirm competitive binding of the ACE2 receptor. The difference was that the binding affinity of the recombinant human ACE2 receptor and the recombinant SARS-CoV-2 spike protein RBD was about 10–100 compared with the affinity between mAb and RBD; thus, the ACE2 receptor was only used for step 3, which involved the 2^nd^ antibody binding.

### Simulation Docking

BioLuminate within Schrödinger Suite was used for scFv homology modeling of MG1141A using the structure of anti-PD1 (PDB code: 6JJP) and anti-SIRPα antibodies (PDB code: 6 NMR) as templates for heavy and light chains, respectively (33). Model quality was assessed using a Ramachandran plot and protein report. Docking of scFv structures of MG1141A to RBD (PDB code: 6M0J) was performed using PIPER in BioLuminate (34). The distances between Lys440 and Thr500 of RBD and complementarity-determining region (CDR) loops were constrained between 2 Å and 10 Å based on the results of epitope binning. BioLuminate suggested 30 best complexes with 70,000 possible protein-protein configurations. The final complexes for MG1141A were selected according to the cluster size and agreement with the epitope binning results; then, energy minimization was performed.

## Results

### Mouse Immunization and Selection of Anti-RBD Antibodies

**Figure 1:**
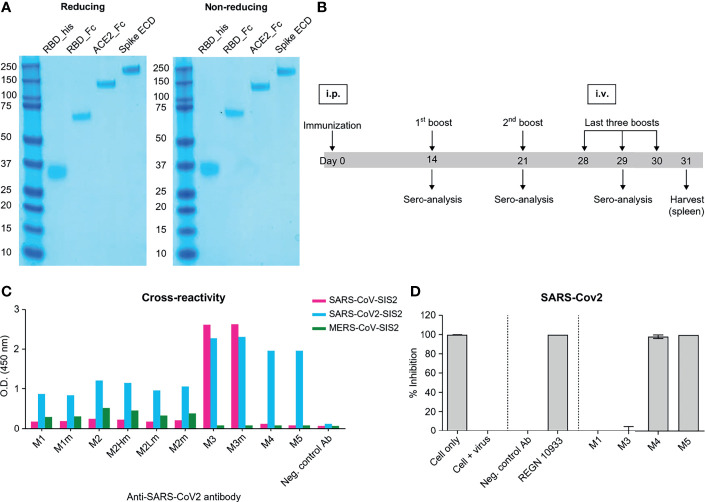
Mouse immunization and *in vitro* screening of anti-SAR-CoV-2 chimeric mAbs. **(A)** SDS-PAGE of recombinant proteins purified from Expi293F cells. SDS-PAGE was performed in reducing (left) and non-reducing conditions (right). All the proteins were expressed as monomers; the same band patterns were observed in both conditions. **(B)** A schematic of the mouse immunization protocol. Immunization, 1^st^, and 2^nd^ booster doses were administered by intraperitoneal injections of 100 µg of RBD mono-Fc. Last three booster doses were injected intravenously with 50 μg of RBD mono-Fc. **(C)** Binding analysis of chimeric mAbs against beta-coronavirus spike proteins. M3, M4, and M5 clones significantly bind to the SARS-CoV-2 spike protein. The M3 clone was cross-reactive to both SARS-CoV and SAR-CoV-2. **(D)** Inhibition screening against SARS-CoV-2 pseudovirus for chimeric mAbs. Each mAb was tested by luciferase assay system. The inhibition of SARS-CoV-2 pseudovirus by the antibodies in human ACE2-overexpressed HEK293 cells was measured in luciferase units. Anti-CEACAM1 antibody is the isotype control.

To prepare soluble antigens for antibody screening, the RBD of SARS-CoV-2 S protein (residues 319–541 aa) and human ACE2 fused to C-terminal human monomeric-Fc (30), named RBD-Fc and ACE2-Fc, respectively, were expressed in Expi293F cells. RBD linked with 6x-His tags at the C-terminal, referred to as RBD-His, was expressed in Expi293F cells. RBD-Fc and ACE2-Fc were purified using a protein-A column. RBD-His was purified using a Ni-NTA column. The size and purity of the purified protein were confirmed by SDS-PAGE under reducing and non-reducing conditions (**Figure 1A**). To generate anti-SARS-CoV-2 RBD antibodies, four female BALB/c mice were immunized with purified RBD-Fc (**Figure 1B**). Recombinant RBD-Fc was expected to be more stable than RBD-His; the C-terminus was masked so that antibodies specific to the region proximal to RBM are generated. However, in mouse immunization, human Fc antibodies are highly likely to be generated. In order to select mice with enhanced generation of antibodies against the RBD, we evaluated antibody titers in serum samples by ELISA against RBD-His after immunization. Only one mouse exhibited enhanced anti-RBD antibody production, whereas the others generated primarily anti-human Fc antibodies. Note that ~1.67 × 10^8^ splenocytes were harvested after sacrificing the mouse. The cDNA corresponding to the antibody variable domain was synthesized from the total RNA of splenocytes with 5ʹ RACE and mouse germline primer sets (31, 32). Mouse single-chain variable fragment (scFv) phage display libraries were constructed for kappa and lambda chains with a diversity of ~6.0 × 10^7^. After three rounds of immuno-tube panning with RBD-His, RBD scFv binders were generated. The RBD binders were converted into the human IgG1 format and expressed in Expi293F cells. The mouse/human chimeric antibodies, named M4, were selected by binding analysis against the S protein and a pseudovirus neutralization assay (**Figures 1C, D**).

### Generation of Humanized Antibody by CDR Grafting

The framework region sequences of the M4 mouse antibody were identified using the mouse germline sequences IGHV5-6 and IGκV6-32. For the humanization of M4, CDR grafting was performed (35, 36). The human frameworks IGHV3-21 and IGκV3-15 were identified using an IgBLAST search, and the sequences that were highly homologous to those of M4, VH, and VL were selected. IGHV3 and IGκV3 are favorable germline pairs among the repertoire of antibodies (37, 38). A simple transfer of the CDR sequence in the humanization process is not sufficient, as preservation of core residues is critical to retaining the biophysical properties of the humanized antibody. The upper core residues indirectly affect the CDR conformation. Residues of the lower and central core and the charged clusters contribute to the overall stability of the antibody (39, 40). Most of the core-residue sequences were identical between mice and humans. Only four residues of the light chain had different sequences; however, the residues exhibited similar characteristics, with no additional mutations (**Supplementary Figure 1**). Humanized M4 was converted into the human IgG1 format and expressed in Expi293F cells. After purification with a protein-A column, the size and purity of humanized M4 IgG were confirmed by SDS-PAGE (**Supplementary Figure 2**). The constructed humanized M4 antibody was named MG1141A.

### Binding Characterization of the Humanized Antibody

**Figure 2:**
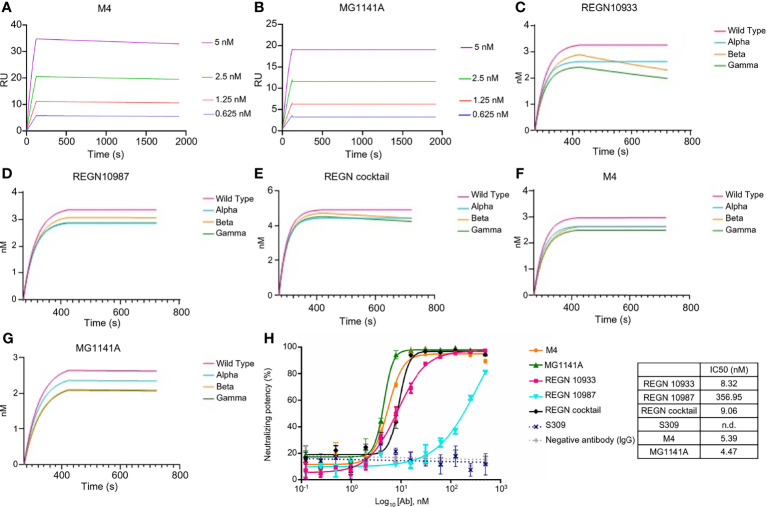
Binding properties of anti-SARS-CoV-2 mAbs. **(A, B)** Binding sensorgrams of SARS-CoV-2 mAbs. Binding analysis of mAbs on a protein-A chip at 25°C using Biacore T-200. S protein was serially diluted (2-fold) from 0.3125 nM to 20 nM and was run on the chip for 180 s for association and 1,800 s for dissociation. Equilibrium dissociation constants (*k*_D_) were calculated from *k*_off_/*k*_on_; at least four concentrations of S proteins were used. **(C–G)** Binding sensorgram of SARS-CoV-2 mAbs against SARS-CoV-2 proteins of alpha (United Kingdom), beta (South Africa), and gamma (Brazil) variants. Binding analysis of RBD variant captured on a Ni-NTA chip using an Octet Qke instrument. SARS-CoV-2 mAbs (125 nM) were run on the chip for 150 s for association and 300 s for dissociation. One representative graph out of two experiments that showed similar results is shown. **(H)** Competitive ELISA of mAbs with RBD against ACE2. Human ACE2 was coated on the ELISA plate, followed by incubation with a pre-incubated mixture of mAbs and RBD at 4°C overnight. The results are represented as a non-linear regression line, fitted by GraphPad Prism 6. The competition of all mAbs was observed, except for S309; the results are represented by dots.

The affinity of the M4 and MG1141A clones was determined by surface plasmon resonance (SPR Biacore T-200). M4 and its humanized form, MG1141A IgGs, bound to the S protein extracellular domain (ECD) of SARS-CoV-2 with a binding affinity of 1.997 × 10^−11^ M and < 1.0 × 10^−12^ M, respectively (**Figures 2A, B**). After humanization, MG1141A exhibited an enhanced dissociation rate (10-fold higher) compared with M4. We evaluated the binding affinity of the mAbs as compared with the potent neutralizing mAbs used in clinical trials such as REGN10933 and REGN10987 of Regeneron Pharmaceuticals and S309 of GSK/Vir (the parent of VIR-7831). The abovementioned mAbs bound to the same antigen with a binding affinity of 3.195 × 10^−11^ M, 1.744 × 10^−11^ M, and < 1.0 × 10^−12^ M, respectively (**Supplementary Figure 3**). Their binding affinity data were as reported in previously published studies (41, 42).

Next, we evaluated the binding of the mAbs against the RBD of alpha (United Kingdom), beta (South Africa), and gamma (Brazil) variants. In this experiment, M4, MG1141A, REGN10933, REGN10987, and REGN cocktail (REGN10933 + 10987, 1:1 mix) antibodies were used. The REGN cocktail was used to compare whether two antibodies with different RBM epitopes were synergistic in their action against variants when used as a mixture (41, 43). As determined by biolayer interferometry assay, REGN10933, an RBM-targeting neutralizing antibody, exhibited a similar binding affinity for the alpha variant as it did for the WT. However, we observed that the binding of REGN10933 to beta and gamma variants was significantly reduced. These results are consistent with previously published data and reconfirmed that mutations in the K417 and E484 residues, which contribute toward the binding of REGN10933 to the RBD, are responsible for the reduced binding. Another RBM-targeting neutralizing antibody, REGN10987, exhibited similar binding affinities with the WT and all the variants. It is possible that the variant used in this test did not harbor the residues K444 or V445, which play an important role in the binding of REGN10987 to the RBD. In contrast, M4 and MG1141A showed no change in binding to the variants. These results suggest that our antibodies may retain their ability to bind to newly generated SARS-CoV-2 variants (**Figures 2C**–**G**). We further evaluated the ability of the antibody to inhibit the binding between the RBD of SARS-CoV-2 and human ACE2. We incubated the RBD with increasing concentrations of each antibody at 4°C overnight and measured its binding to ACE2 at 37°C. The binding of the RBD with ACE2 was effectively inhibited with an IC_50_ at the single-digit nanomolar level by M4, MG1141A, and Regeneron antibodies except S309 (**Figure 2H**). Thus, our antibodies had an extremely high affinity for the S protein and effectively competed with ACE2. Consequently, we expect our antibodies to have high neutralizing potency against SARS-CoV-2.

### MG1141A Exhibits Comparable Neutralizing Efficacy Against SARS-CoV-2 WT and Variants

**Figure 3:**
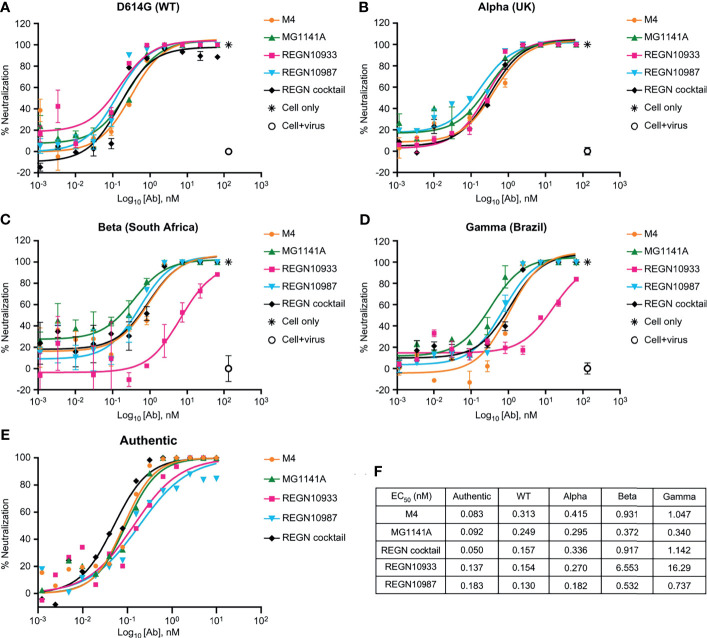
Neutralizing activity of anti-SARS-CoV-2 mAbs. **(A–D)** Dose-dependent neutralization of SARS-CoV-2 pseudovirus of D614G (wild type), Alpha (United Kingdom), Beta (South Africa), and Gamma (Brazil). Neutralizing activities of 11-point–diluted anti-SARS-CoV-2 mAbs were evaluated on ACE2-HEK293 cells using the luciferase assay system. The EC_50_ and EC_90_ values were determined by curve fitting with non-linear regression analysis (sigmoidal dose response). Bars, mean ± standard error of the mean (SEM) of triplicates derived from one of the two experiments. **(E)** Dose-dependent neutralization of SARS-CoV-2 isolated from a patient (Korea/KUMC45/2020, GH clade) by mAbs, as evaluated by a plaque reduction neutralization test on Vero cells. One representative out of three experiments with similar results is shown. **(F)** The EC_50_ values of the *in vitro* neutralizing assay against variant viruses.

We performed authentic virus and pseudovirus neutralization assays using a lentiviral system expressing the SARS-CoV-2 spike proteins and SARS-CoV-2 receptor-expressing ACE2-HEK293 stable cell line (**Supplementary Figure 4A**). MG1141A (EC_50_ = 0.249 nM, 95% confidence interval [CI] 0.150–0.412) showed a neutralizing efficacy similar to that of the original clone, M4 (EC_50_ = 0.313 nM, 95% CI 0.142–0.688). The neutralization potency was comparable to that of single Regeneron antibodies (REGN10933, EC_50_ = 0.154 nM, 95% CI 0.077–0.307 or REGN10987, EC_50_ = 0.130 nM, 95% CI 0.085–0.198) or a cocktail (EC_50_ = 0.157 nM, 95% CI 0.087–0.283) of Regeneron antibodies against D614G (WT) pseudovirus (**Figure 3A**). Next, to assess the neutralizing efficacy of MG1141A against SARS-CoV-2 spike variants, alpha (United Kingdom), beta (South Africa), and gamma (Brazil) pseudovirus neutralization assays were performed using a lentiviral system expressing each SARS-CoV-2 spike variant (**Supplementary Figure 5**). M4 and MG1141A were able to neutralize three variant pseudoviruses with a neutralizing efficacy comparable to that of REGN10987 or Regeneron cocktail (**Figures 3B**–**D**). REGN10933 failed to mediate 100% neutralization of beta or gamma variant pseudovirus infection in cells even at high concentrations (**Figures 3C, D**). Taken together, our humanized SARS-CoV-2 antibody candidate, MG1141A, potently neutralized WT and selected spike-mutant SARS-CoV-2 pseudovirus particles, and its neutralizing efficacy is comparable to that of the Regeneron cocktail. We determined both the EC_50_ and EC_90_ values of MG1141A against clinical SARS-CoV-2 isolates (Korea/KUMC45/2020, clade GH) by the PRNT. M4 and MG1141A showed neutralization potency against the authentic virus with EC_50_ values of 0.083 nM (95% CI 0.053–0.121) and 0.092 nM (95% CI 0.059–0.137), respectively. Moreover, MG1141A showed strong neutralizing activity, with an EC_90_ value of 0.559 nM (95% CI 0.251–1.202), which was comparable to that of the Regeneron cocktail (EC_90_ = 0.346 nM, 95% CI 0.169–0.685) (**Figure 3E**). The EC_50_ values of the in vitro neutralizing assay of anti-SARS-CoV-2 mAbs against variant viruses (**Figure 3F**).

### MG1141A Has the Potential to Induce Fc-Mediated Antibody Effector Functions

**Figure 4:**
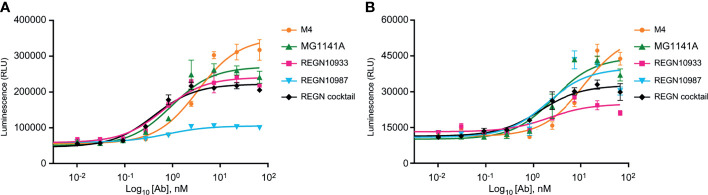
Fc-mediated effector functions of anti-SARS-CoV-2 mAbs. **(A)** Activation of ADCC signaling was evaluated using a FcγRIIIa-expressing reporter cell line as effector cells and SARS-CoV-2 S-glycoprotein-expressing HT1080 as target cells. **(B)** Activation of ADCP signaling was measured using FcγRIIa-H-expressing reporter cell line as effector cells and SARS-CoV-2 S-glycoprotein-expressing HT1080 as target cells. In **(A, B)**, bars are mean ± standard error of the mean (SEM) of duplicates.

The neutralizing antibody mediates additional antiviral functions to accelerate the clearance of virus particles and infected cells through Fc gamma receptor (FcγR)-mediated effector functions such as ADCC and ADCP (44–46). To assess the Fc receptor-dependent function of anti-SARS-CoV-2 antibody candidates, ADCC and ADCP assays were performed in Jurkat reporter cells and SARS-CoV-2 S protein-expressing HT1080 target cells (**Supplementary Figure 4B**). We found that MG1141A (EC_50_ = 0.943 nM, 95% CI 0.514–1.728) induced ADCC activity against SARS-CoV-2 S protein-expressing targets higher than that of the original mouse clone M4 (EC_50_ = 3.376 nM, 95% CI 2.060–5.532) and similar to that of the single Regeneron antibody (REGN10933, EC_50_ = 0.643 nM, 95% CI 0.382–1.083 or REGN10987, EC_50_ = 0.750 nM, 95% CI 0.427–1.317) or cocktail (EC_50_ = 0.406 nM, 95% CI 0.264–0.625) of Regeneron antibodies (**Figure 4A**). MG1141A induced ADCP activity (EC_50_ = 2.644 nM, 95% CI 1.208–5.786) comparable to that of a single Regeneron antibody (REGN10933, EC_50_ = 2.112 nM, 95% CI 0.519–8.595 or REGN10987, EC_50_ = 1.949 nM, 95% CI 0.663–5.730) or a cocktail (EC_50_ = 1.353 nM, 95% CI 0.792–2.309) of Regeneron antibodies (**Figure 4B**). Collectively, the data in **Figure 4** show that MG1141A has the potential to induce the Fc-mediated antibody effector functions, ADCC and ADCP, to mediate maximal therapeutic efficacy.

### Epitope Binning of MG1141A

**Figure 5:**
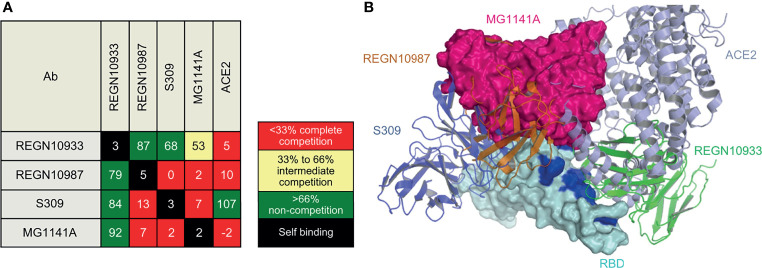
Epitope binning of MG1141A. **(A)** Epitope binning heat map. Based on the reference binding, the binding level of the 2^nd^ antibody is shown in percentages (%) and color. The 1^st^ binding antibody is shown in the row, while the 2^nd^ binding antibody is shown in the column. The diagonal lines in white indicate that the self-binding of 1^st^ and 2^nd^ binding antibodies is identical. **(B)** Superposition of MG1141A scFv docking model (hot pink) on RBD structures (cyan) complexed with REGN10987, REGN10933, and S309. MG1141A scFv collided with REGN10987 (orange), S309 (tv blue), and ACE2 (light blue), but not with REGN10933 (green). The ACE2-binding region is represented by the blue color.

Biolayer interferometry (BLI), a label-free detection system, was used for epitope binning of MG1141A. BLI, which can detect real-time competitive binding of antibodies, requires mAbs that have been identified as competitors. RBD-His was immobilized using an anti-penta-His biosensor, and the mAb was sequentially bound in an in-tandem manner. This allowed us to estimate the epitope of the new clone. To predict the epitope of MG1141A, three control antibodies with different epitopes were used: (1) REGN10933, which interacts with the ACE2 binding site, (2) REGN10987, which binds to an alternative ACE2 binding site; and (3) S309, which has an ACE2 non-binding site as an epitope different from those of previous Regeneron antibodies (42, 43). The epitope binning results showed that MG1141A competes with REGN10987 and S309. Against REGN10933, MG1141A showed intermediate competitive binding, whereas REGN10987 and S309 did not compete. Additionally, we determined the competitive binding of MG1141A and the ACE2 receptor to the target protein. Unlike S309, which did not compete with ACE2, MG1141A exhibited complete competition with ACE2. Overall, these results suggest that MG1141A exhibited full competition with the ACE2 receptor for binding to the target protein and possessed a significantly different epitope from those of the control Abs included in previous studies (**Figure 5A** and **Supplementary Figure 6**).

### Simulation Docking of MG1141A With RBD

We performed homology antibody modeling and docking for the RBD of the viral S protein. BioLuminate suggested 15 models, and the first and second large clusters had 185 and 138 docking models, respectively. However, because the models from these clusters did not block the binding of ACE2, REGN10987, and S309 simultaneously, they were discarded. According to the binning results, MG1141A inhibited the binding of ACE2, REGN10987, and S309 to the target protein. The docking model from the third large cluster, which was 118 residues in size, was selected because it satisfied these criteria (**Figure 5B**). The sizes of the other clusters were less than 90 and did not satisfy these criteria. The selection criteria could be subjective; nevertheless, the docking model should be consistent with the binning results, and we believe our model is a suitable representation of the binning results. The residues of MG1141A did not interact with the mutated residues of the recently emerged variant viruses, except for Asn501Tyr. The mutation of Asn501 into Tyr501 did not have a negative effect on RBD binding because of the newly generated π-π interactions between Tyr56 and Tyr501. MG1141A did not bind to the SARS-CoV-2 RBD (**Figure 1**). According to our model, the binding affinity of MG1141A to the RBD of SARS-CoV-2 may be reduced because of substitutions of Ser373 and Asn439 to Phe373 and Arg439 in the epitope region of SARS-CoV-2.

## Discussion

Antibody therapeutics against various viruses are being studied, and several antibodies have been approved for phase III clinical trials, including those that confer passive immunity to RSV (47, 48), reduce the mortality of Ebola virus infections (49), and reduce hospitalization in patients with COVID-19 infections. There are various methods for generating antibody candidates, such as from the blood of convalescent donors. An *in vitro* library using antibody genes from human B cells has also been constructed for this purpose. Approximately 10 years ago, procedures such as rapid isolation of antigen-positive B cells and immune profiling based on high-throughput sequencing were developed (50). These approaches, as well as versatile B cell repertoire analysis techniques, have been employed in the development of COVID-19 antibody therapeutics (51–53).

Humans produce antibodies specific to hyper-immunogenic regions of viral proteins or regions that effectively inhibit the binding of these proteins to human receptors. Viruses respond by introducing mutations in the regions against which human antibodies are formed to overcome the immune response or by evolving into strains that exhibit enhanced infection efficiency and survival (54–56). In the case of SARS-CoV-2, mutations in the non-structure proteins coded by ORF1a and ORF1b occurred initially. Subsequently, S protein mutations such as D614G caused increased infectivity. N439K and N501Y mutations, which confer the capability of infectivity and immune evasion to SARS-CoV-2, were also detected in SARS-CoV-2. Most of the antibody therapeutics and vaccines currently being developed target S proteins; thus, SARS-CoV-2 can accelerate its evolution to escape human immune responses, thereby reducing the efficacy of SARS-CoV-2 treatments.

Our objective was to develop an antibody that can effectively neutralize the recently detected SARS-CoV-2 mutations and imminent mutations that may be potentially deleterious to human health. We generated the initial candidate antibodies through mouse immunization among various antibody screening methods. Currently, human antibodies are produced in transgenic mice using human antibody genes, which have been frequently used for mouse immune screening. REGN10933 (casirivimab) and REGN10987 (imdevimab), the COVID-19 antibodies produced by Regeneron Pharmaceuticals, are derived from transgenic mice (43). However, the source of antibody genes is the human antibody repertoire, providing these antibody candidates with the potential to reduce the severity of SARS-CoV-2 variant infections. Therefore, we decided to generate an initial candidate antibody by immunizing normal mice and then generating a final antibody through humanization. To generate an effective neutralizing antibody, the RBD, which is the ACE2-binding region of the S protein, was used as an antigen for mouse immunization. The stability of the RBD was enhanced by fusing it to the Fc region. Balb/c mice were immunized with RBD-Fc three times by intraperitoneal and intravenous injections. The scFv phage display library was constructed using the immune repertoire of immunized mice. The library was panned three times with the S protein coated on an immuno-tube. The obtained scFv clones were converted into human IgG1-kappa to produce an anti-RBD chimeric IgG1 antibody, which were expressed in Expi293F cells. The M4 clones exhibited optimal IC_50_ values and were selected as initial candidates. The M4 sequence was identified using the mouse germline sequences IGHV5-6 and IGKV6-32. They showed high homology with human IGHV3-21 and IGKV3-15, and a humanized antibody was prepared through CDR grafting. We generated several humanized clones through CDR grafting, and an *in vitro* assay demonstrated that the abovementioned clone was the most efficacious (data not shown). In general, the binding affinity of the antibodies tended to decrease after humanization, but the K_D_ value of MG1141A (M4 humanized clone) was maintained at 20 pM. The overall structure of the variable region of the humanized antibodies was not affected due to the high homology between the frameworks. Moreover, the core residues were preserved, and the affinity of the humanized antibodies was maintained by the canonical structure of the CDRs. For comparative analysis of its efficacy, REGN10933 and REGN10937 of Regeneron Pharmaceuticals, which are currently in phase III clinical trials, were used as controls. MG1141A exhibited an IC_50_ of 92 pM. This was equivalent to the neutralizing effect exhibited by REGN antibodies in authentic virus and pseudovirus neutralization assays. Furthermore, MG1141A induced both ADCC and ADCP, similar to REGN. As a result, MG1141A showed equivalent *in vitro* potency as a single monoclonal antibody, compared with the REGN cocktail. Furthermore, it binds to a different epitope and may serve as a novel antibody therapeutic agent.

We also generated human monoclonal antibodies from the blood of the COVID-19 convalescent donors. The blood samples were collected within 1 month of recovery from 19 donors who were infected with COVID-19 between March and May 2020 (IRB number: YUMC 2020-04-009). For donor screening, antibody titers in the blood were measured by ELISA against the S protein ECD and RBD, and the PRNT test was performed on the authentic virus (**Supplementary Figure 7**). Among the 19 donors, the B cell repertoire gene was obtained from memory B cells from 8 donors who showed a high antibody titer and neutralizing effect to construct a phage display library. The phage display library screening was performed as described in this manuscript. A total of 99 unique antibody sequences were generated, of which 28 clones showing high binding were purified with IgG1 to assess the affinity and neutralizing effect. Fully human monoclonal antibodies bound to the S protein ECD at sub-nM to single-digit nM levels, but there were five clones that specifically bound to the RBD. Eight clones confirmed neutralizing efficacy at the level of double- to triple-digit nM against authentic virus (**Supplementary Table 2**). We compared the efficacy of fully human monoclonal antibodies and M4 clones and confirmed that M4 had the best efficacy. Regeneron also proceeded to generate antibodies from the mouse immune method and the blood of the convalescent donors. The antibody pool generated through mouse immunization was superior to the antibody pool from humans, and as such, casirivimab and imdevimab could be generated through mouse immunization (41). The method of generating antibodies from convalescent donors has the advantage generating antibodies against SARS-CoV-2 most quickly; therefore, it was used in the early stages of the development of COVID-19 antibody therapeutics. However, because the production of antibodies by immune response after SAR-CoV-2 infection in humans occurs in many hyper-immunogenic sites of the S protein ECD, it was not easy to find effective neutralizing antibodies because only a few bind specifically to the RBM. In the case of RSV and influenza, because infections can occur several times over the human lifespan, many effective neutralizing antibodies can be generated, but in the case of single-time infections such as SARS-CoV-2, designing RBD antigens to bind specifically to the RBM and generating antibodies through mouse immunization is considered to be one way to generate more effective neutralizing antibodies than fully human antibodies from convalescent human donors.

## Data Availability Statement

The original contributions presented in the study are included in the article/**Supplementary Material**. Further inquiries can be directed to the corresponding author.

## Ethics Statement

The animal study was reviewed and approved by Institutional Animal Care and Use Committee of GC Pharma. The collection of convalescent human blood was reviewed and approved by Institutional Review Board of Yeungnam University.

## Author Contributions

SL, SJ, and D-SK conceived and designed the project. SL, SJ, JKa, SP, YH, HN, MK, JL, JKi, MO, JR, JS, YK, and D-SK performed and designed experiments. YH, JL, M-SP, Y-SK, HP, and D-SK provided advice on experimental design and data analysis. SL, SJ, HN, MK, KC, JKi, and D-SK wrote the manuscript. All authors contributed to the article and approved the submitted version.

## Funding

This research was supported by the Mogam Institute for Biomedical Research and GC Pharma, Republic of Korea (Grant number: MG1141A). The funder was not involved in the study design, collection, analysis, interpretation of data, the writing of this article or the decision to submit it for publication.

## Conflict of Interest

SL, SJ, JKa, SP, YH, HN, MK, JL, KC, MO, JR, JL, and D-SK submitted a patent application on neutralizing antibodies against SARS-CoV-2.

The remaining authors declare that the research was conducted in the absence of any commercial or financial relationships that could be construed as a potential conflict of interest.

## Publisher’s Note

All claims expressed in this article are solely those of the authors and do not necessarily represent those of their affiliated organizations, or those of the publisher, the editors and the reviewers. Any product that may be evaluated in this article, or claim that may be made by its manufacturer, is not guaranteed or endorsed by the publisher.
