# Supplementary material for: MG1141A as a Highly Potent Monoclonal Neutralizing Antibody Against SARS-CoV-2 Variants
Source: Front Immunol. 2021 Nov 18;12:778829. doi: 10.3389/fimmu.2021.778829 (PMC8637776; doi:10.3389/fimmu.2021.778829)
Supplement: Supplementary file 1 [file DataSheet_1.docx]

***Supplementary Material***


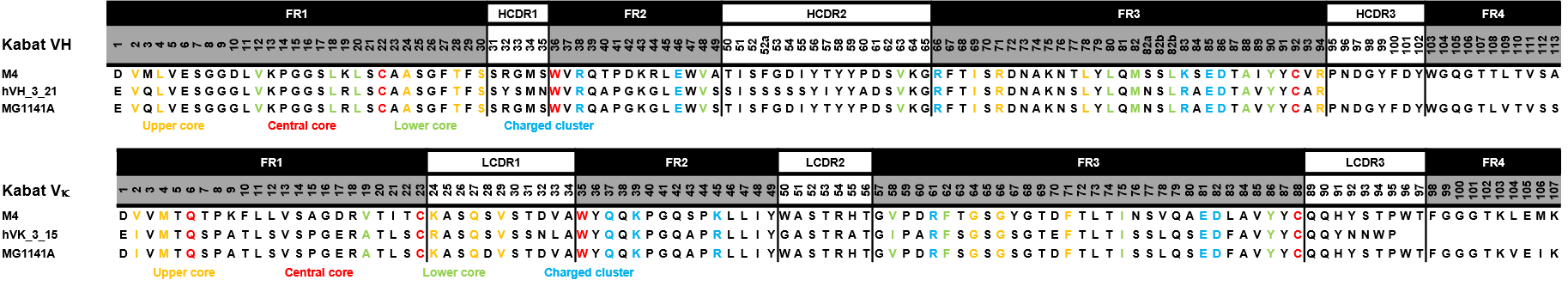


**Supplementary Figure 1. Amino-acid sequence alignment between mouse and humanized variable domains.** Mouse CDR sequences were grafted onto human VH3-21 and Vk3-15 germline sequences. The sequences are numbered according to Kabat numbering. Core residues are marked by different colors: upper core (orange), central core (red), lower core (green), and charged cluster (blue).


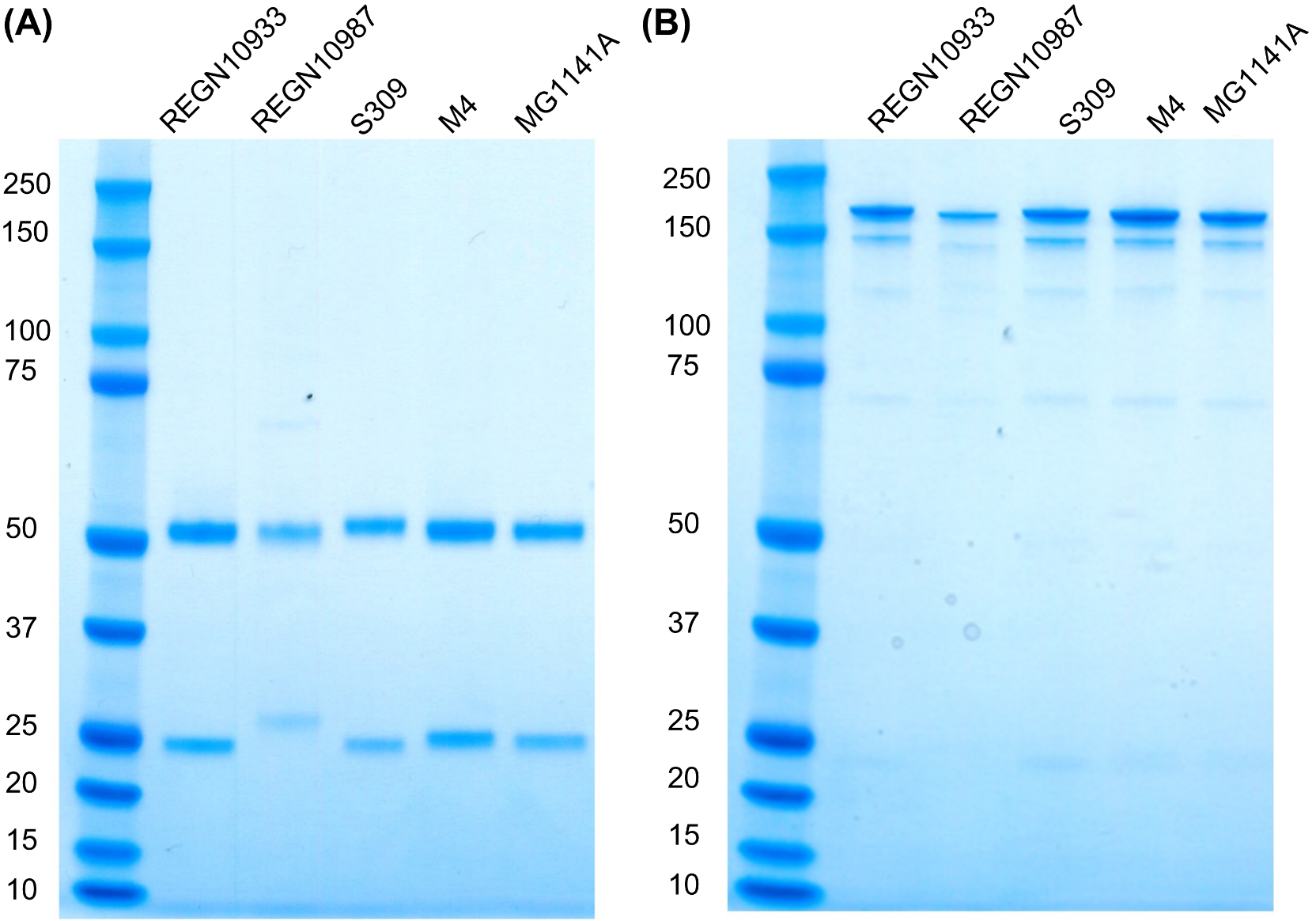


**Supplementary Figure 2. SDS-PAGE results of the purified antibodies.** The purity of in-house antibodies was verified using SDS-PAGE. The clone names of the antibodies are indicated above the SDS-PAGE gel. SDS-PAGE was performed under reducing (A) and non-reducing (B) conditions.


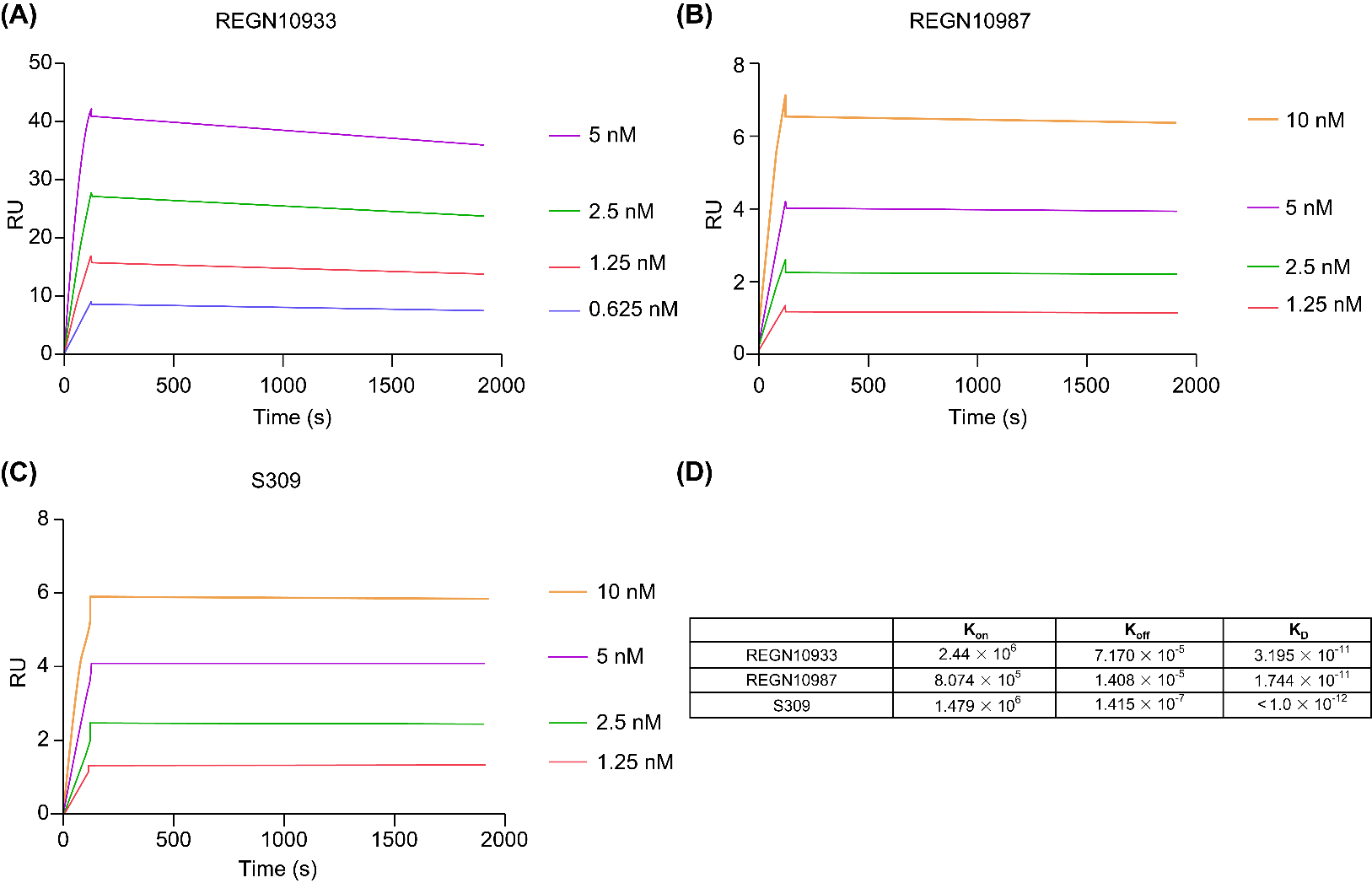


**Supplementary Figure 3. Binding characterization of anti-SARS-CoV-2 mAbs used in clinical trials.** Binding analysis of mAbs captured on a protein-A chip at 25 °C using Biacore T-200. S protein serial diluted (2-fold) from 0.3125 nM to 20 nM was run on the chip for 180 sec for association and 1,800 sec for dissociation. Equilibrium dissociation constants (K_D_) were calculated from K_off_/K_on_. At least four concentrations of S proteins were used.





**Supplementary Figure 4. A** Representative flow-cytometry plots of ACE2 expression from the ACE2-HEK293 stable cell line. **B** Representative flow cytometry of SARS-CoV-2 Spike protein expression from the HT1080-S stable cell line.


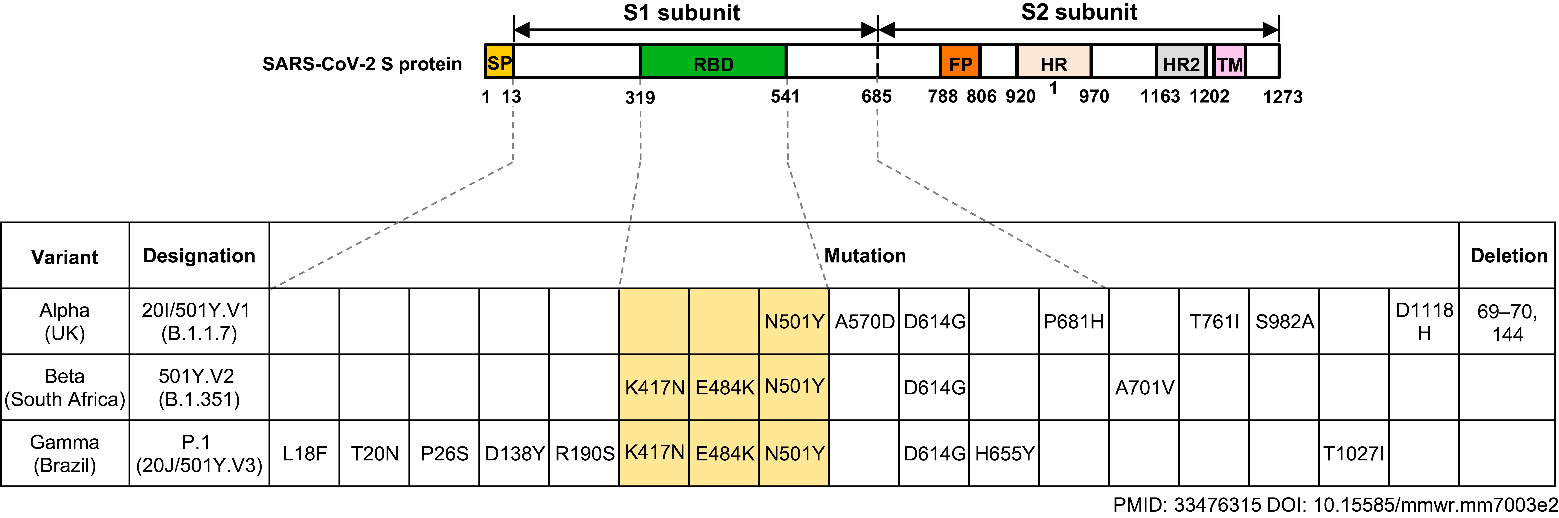


**Supplementary Figure 5. Schematic figure of mutation sequences of SARS-CoV-2 variants.** The SARS-CoV-2 S protein consists of two subunits (S1 and S2). The S1 subunit contains a receptor-binding domain (RBD), and the S2 subunit contains a fusion peptide (FP), two heptad repeats (HR1 and HR2), and a transmembrane region. Each SARS-CoV-2 pseudovirus variant was generated with mutated representative sequences in the S protein. The UK variant additionally had the deletion of residues 69-70 and 144. The mutations are located in the receptor-binding motif (RBM) and are highlighted by yellow boxes in the mutated sequences.


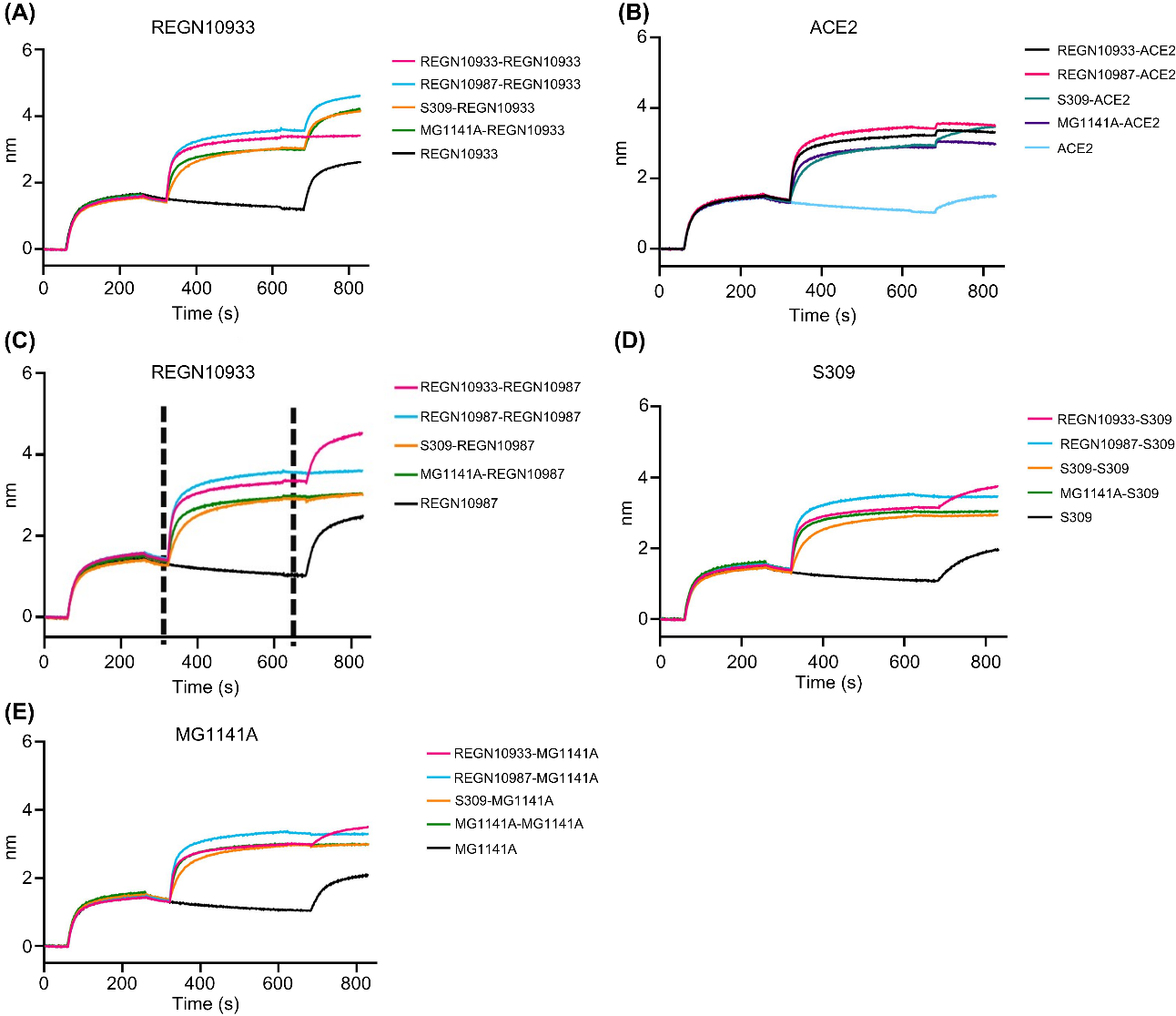


**Supplementary Figure 6. Epitope binning of MG1141A.** Trace data of epitope binning performed using the in-tandem method. The steps of antigen immobilization, 1^st^ association, and 2^nd^ association are shown by a dashed line. Among the traces, the 2^nd^ binding trace was observed only in the reference trace. Based on this, the binding percentages were classified into 3 stages: < 33%, complete competition; 33%–66 %, intermediate competition; > 66 %, no competition.

**
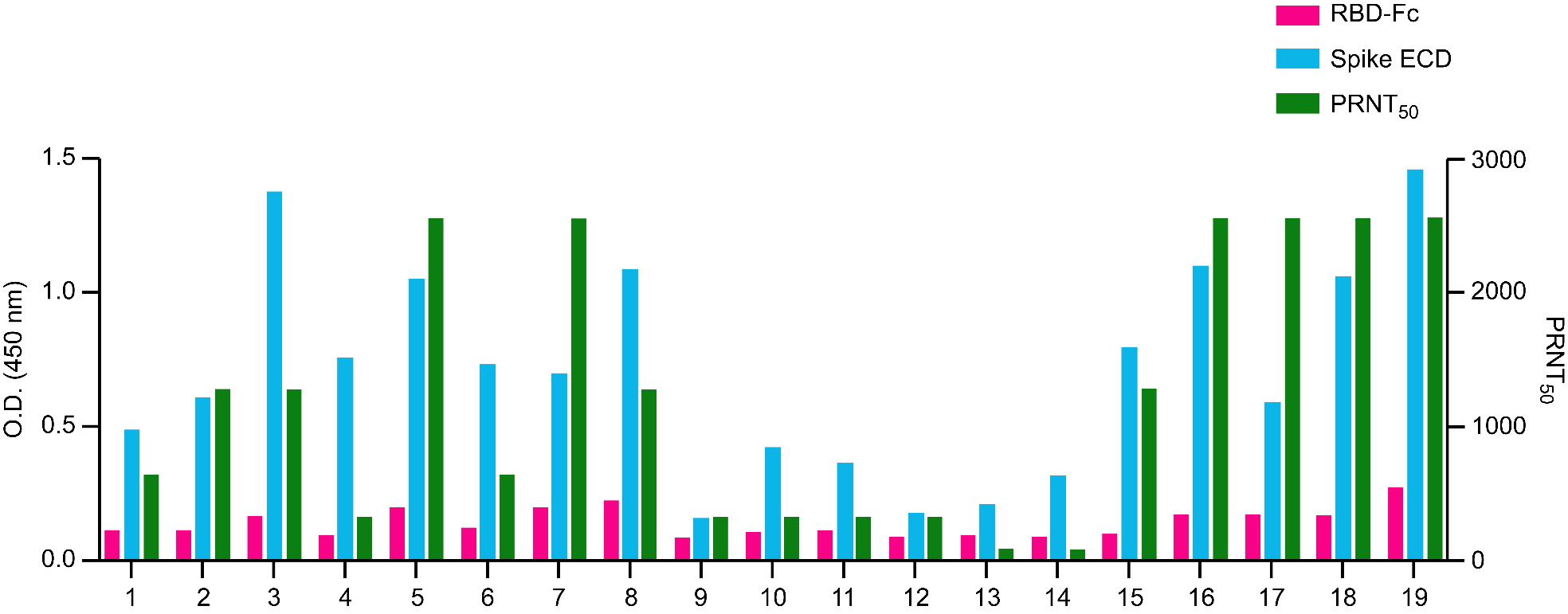
**

**Supplementary Figure 7. Donor screening.** Human sera and peripheral blood mononuclear cells (PBMCs) were collected and isolated from the 19 COVID-19 convalescent donors. Serum was separated from the collected blood and the antibody titer was analyzed against the S protein extracellular domain (ECD) and receptor-binding domain (RBD)-Fc. A 96-well ELISA plate was coated with S protein ECD and RBD protein at 0.15 µg in each well in phosphate-buffered saline (PBS) overnight at 4 °C. After washing with 0.05% Tween 20 in PBS (PBST) and blocking with 5% bovine serum albumin in PBS, the plasmas were incubated for 2 h at room temperature. The plasma from donors was diluted 1:100. After washing with PBST, anti-human Fc-HRP (1:1000) was used as the secondary antibody. Tetramethylbenzidine solution and stop solution were used for detection. Antibody titers showed higher binding to the S protein ECD than to RBD-Fc. Antibody titers were slightly correlated with the median plaque reduction neutralization result (PRNT_50_). Donor numbers 3, 5, 7, 8, 16, 17, 18, and 19 were chosen to construct the phage display library. The PRNT_50_ method is included in the Materials and Methods.

**Supplementary Table 1.** **Information on RBD variant proteins**

| **Variant** | **Name** | **Mutation** | **Cat. no.** |
| --- | --- | --- | --- |
| WT | SARS-CoV Spike/RBD Protein (RBD, His Tag) | - | Sino Biologics, 40150-V08B2 |
| UK variant \| B.1.1.7 | SARS-CoV-2 (2019-nCoV) Spike RBD(N501Y)-His Recombinant Protein | N501Y | Sino Biologics, 40592-V08H82 |
| South Africa variant \| B.1.351 | SARS-CoV-2 (2019-nCoV) Spike RBD(K417N, E484K, N501Y)-His Recombinant Protein | K417N, E484K, N501Y | Sino Biologics, 40592-V08H85 |
| Brazil variant \| P.1 | SARS-CoV-2 (2019-nCoV) Spike RBD (K417T, E484K, N501Y) Protein (His Tag) | K417T, E484K, N501Y | Sino Biologics, 40592-V08H86 |

**Supplementary Table 2. Screening results of anti-SARS-CoV-2 chimeric and fully human antibodies**

| **Origin** | **Clone name** | **Germline^a^** | | **EC_50_, nM^b^** | **Affinity (*k*_D_, nM)^c^** | |
| --- | --- | --- | --- | --- | --- | --- |
|  |  | **Heavy chain** | **Light chain** |  | **RBD** | **S protein ECD** |
| **Mouse**  **chimeric Ab** | M4 | IGHV 5-6 | IGKV 6-32 | 0.08 | 0.263 | 0.107 |
| **Human Ab** | 2 | IGHV3-23 | IGKV1-33 | >100 | 0.459 | 0.37 |
|  | 3 | IGHV3-23 | IGKV1-33 | >100 | 0.468 | 0.323 |
|  | 4 | IGHV3-23 | IGKV1-33 | >50 | 0.45 | 0.381 |
|  | 5 | IGHV3-23 | IGKV1-33 | >50 | 0.71 | 0.259 |
|  | 8 | IGHV3-48 | IGLV1-44 | n.d. | n.d. | 0.626 |
|  | 10 | IGHV3-48 | IGLV1-44 | n.d. | n.d. | 0.265 |
|  | 11 | IGHV3-7 | IGLV2-11 | n.d. | n.d. | 0.44 |
|  | 13 | IGHV3-30 | IGLV1-40 | >100 | n.d. | 0.207 |
|  | 14 | IGHV3-7 | IGLV1-40 | >100 | n.d. | 0.119 |
|  | 15 | IGHV3-23 | IGLV1-50 | n.d. | n.d. | 0.34 |
|  | 16 | IGHV3-23 | IGLV1-40 | n.d. | n.d. | 0.996 |
|  | 17 | IGHV3-7 | IGLV1-40 | >100 | n.d. | 0.373 |
|  | 18 | IGHV3-23 | IGLV1-40 | n.d. | n.d. | 0.293 |
|  | 21 | IGHV3-7 | IGLV1-40 | n.d. | n.d. | 0.453 |
|  | 22 | IGHV3-7 | IGLV1-40 | n.d. | n.d. | 0.904 |
|  | 23 | IGHV3-7 | IGLV1-40 | n.d. | n.d. | 1.825 |
|  | 25 | IGHV3-7 | IGLV1-40 | n.d. | n.d. | 0.99 |
|  | 27 | IGHV3-7 | IGLV1-40 | n.d. | n.d. | 1.55 |
|  | 28 | IGHV3-7 | IGKV1-39 | n.d. | n.d. | 0.632 |
|  | 29 | IGHV3-7 | IGLV1-40 | n.d. | n.d. | 1.16 |
|  | 30 | IGHV3-7 | IGLV1-40 | n.d. | n.d. | 2.43 |
|  | 31 | IGHV3-7 | IGLV1-40 | n.d. | n.d. | 1.481 |
|  | 32 | IGHV3-7 | IGLV1-40 | n.d. | n.d. | 3.216 |
|  | 33 | IGHV3-7 | IGKV1-39 | >100 | n.d. | 0.111 |
|  | 35 | IGHV3-9 | IGLV2-14 | n.d. | 1.104 | 0.667 |
|  | 38 | IGHV1-46 | IGLV1-51 | n.d. | n.d. | 1.095 |
|  | 41 | IGHV4-4 | IGKV1-39 | n.d. | n.d. | 0.473 |
|  | 42 | IGHV3-48 | IGLV3-21 | n.d. | n.d. | 2.177 |
| **Control Ab** | REGN10933 | IGHV3-11 | IGKV1-33 | 0.08 | 0.466 | 0.359 |
|  | REGN10987 | IGHV3-30 | IGLV2-14 | 0.08 | 0.534 | 0.493 |

^a^ Antibody germline sequences were identified using an IgBLAST search.

^b^ The EC_50_ values of antibodies against clinical SARS-CoV-2 isolates (Korea/KUMC45/2020, clade GH) were determined by the plaque reduction neutralization test (PRNT).

^c^ The affinity of antibodies was determined by ELISA.
